# Haplotypic polymorphisms and mutation rate estimates of 22 Y-chromosome STRs in the Northern Chinese Han father–son pairs

**DOI:** 10.1038/s41598-018-25362-3

**Published:** 2018-05-08

**Authors:** Yaran Yang, Weini Wang, Feng Cheng, Man Chen, Tong Chen, Jing Zhao, Chong Chen, Yan Shi, Chen Li, Chuguang Chen, Yacheng Liu, Jiangwei Yan

**Affiliations:** 1 CAS Key Laboratory of Genome Sciences and Information, Beijing Institute of Genomics, Chinese Academy of Sciences, Beijing, 100101 P.R. China; 2 Forensic Science Center of ShenZhen City, Guangdong, 518040 P.R. China; 3 Shanxi Medical University, Taiyuan, 030009 P.R. China; 4 University of Chinese Academy of Sciences, Beijing, 100049 P.R. China; 5 Beijing Tongda Shoucheng Institute of Forensic Science, Beijing, 100085 P.R. China; 6 Beijing Microread Genetics Co., Ltd, Beijing, 100044 P.R. China

## Abstract

Y chromosome Short tandem repeats (Y-STRs) analysis has been widely used in forensic identification, kinship testing, and population evolution. An accurate understanding of haplotype and mutation rate will benefit these applications. In this work, we analyzed 1123 male samples from Northern Chinese Han population which including 578 DNA-confirmed father-son pairs at 22 Y-STRs loci. A total of 537 haplotypes were observed and the overall haplotype diversity was calculated as 1.0000 ± 0.0001. Except that only two haplotypes were observed twice, all the rest of the 535 were unique. Furthermore, totally 47 mutations were observed during 13,872 paternal meiosis. The mutation rate for each locus estimates ranged from 0.0 to 15.6 × 10^−3^ with an average mutation rate 3.4 × 10^−3^ (95% CI 2.5–4.5 × 10^−3^). Among the 22 loci, DYS449, DYS389 II and DYS458 are the most prone to mutations. This study adds to the growing data on Y-STR haplotype diversity and mutation rates and could be very useful for population and forensic genetics.

## Introduction

Y chromosome Short tandem repeats (Y-STRs) are widely used in genetic epidemiology^1^, forensic genetics^2^ and human migration^3^ because of its paternal inheritance and human population structuring^4^. However, just the same as autosomal STR, Y-STRs also have high mutation rates^5^. Therefore, reliable estimates of mutation rates of Y-STRs are a prerequisite for the accurate application based on Y-STR analysis. Several studies on estimating Y-STR mutation rates had been reported, such as investigating the father–son pairs from confirmed paternity^6^, male individuals from deep-rooted pedigrees^7^, genotyping sperm cells^8^, and using Y-STR population data with known history^9^. Of these approaches, estimating Y-STR mutation rates through the direct observation of allelic transmission between father and son is the most accurate, as long as large numbers of meiosis could be investigated.

In this study, We determined the haplotypes and mutation rates for the 22 Y-STRs, DYS19, DYS385a/b, DYS388, DYS389I/II, DYS390, DYS391, DYS392, DYS393, DYS437, DYS438, DYS439, DYS444, DYS447, DYS448, DYS449, DYS456, DYS458, DYS522, DYS527a/b, DYS635 and Y-GATA-H4 in 1123 Northern Chinese Han male individuals from 578 father–son pairs.

## Materials and Methods

### Samples and DNA extraction

Blood samples were collected from 1123 healthy Northern Chinese Han male individuals. Among these individuals, there had 578 father–son pairs. All father–son pairs were confirmed by using autosomal STRs typing based on 39 autosomal STRs by using Microreader^TM^ 21 ID and 23 SP system (Microread Genetics Incorporation, China), with a minimum paternity probability of 99.99%. All individuals signed the informed consent before participating in this study. Genomic DNA was extracted using Chelex resin method^10^. The quantity of DNA was quantified by Qubit® Quantitation System (Invitrogen, CA, USA). All experiments of this study were carried out in accordance with the guidelines and regulations of the Ethical Committee of Beijing Institute of Genomics, Chinese Academy of Sciences (Protocol name: A study on the Haplotypic polymorphisms and mutation rate estimates of Y-chromosome STRs in father–son pairs. No. 2016033).

### Multiplex PCR amplification and genotyping

The samples were amplified 22 Y-STR loci (DYS19, DYS385a/b, DYS388, DYS389I/II, DYS390, DYS391, DYS392, DYS393, DYS437, DYS438, DYS439, DYS444, DYS447, DYS448, DYS449, DYS456, DYS458, DYS522, DYS527a/b, DYS635 and Y-GATA-H4) using AGCU™ Y24 Plus amplification kit (AGCU ScienTech Incorporation, Wuxi, China) following manufacturer’s recommendations. All the PCR amplification was proceeded respectively in an ABI PRISM^®^ GeneAmp^®^ 9700 thermal cycler. PCR products were detected on ABI PRISM^®^ 3130xl Genetic Analyzer according to the manufacturer’s recommendations. Electrophoretic result was analyzed using GeneMapper^®^ ID-X software.

### Quality control

A male DNA sample 9948 (Promega Corporation, WI, USA) and female DNA sample 9947 A (Promega Corporation) were used as reference and negative control for each batch of genotyping. All the experiments were carried out at the laboratory accredited by the China National Accreditation Service (CNAS) and strictly followed the recommendations on the analysis of Y-STRs by DNA Commission of the International Society of Forensic Genetics (ISFG)^11^.

### Statistical analysis

Haplotype and allele frequencies were calculated by the gene counting method. Gene diversity (GD) for each locus was calculated using the formula: GD = [n (1 − ∑pi^2^)]/(n − 1), where n is the number of alleles, pi is the frequency of the ith allele. Discrimination capacity (DC) was determined as DC = Ndiff/N, where Ndiff and N was the number of different haplotypes and the sample size, repectivly. Haplotype diversity (HD) and Standard error (SE) was calculated according to Nei’s formula^12^. Mutation rates were calculated as the number of mutations divided by the number of Meiosis. Confidence intervals (CI) were estimated from the binominal standard deviation^13^. In mutation counting, there were two father–son pairs where one-step mutation seen for both DYS389I and DYS389II, for instance (13, 29) → (14, 30). These were treated as one mutation instead of two because DYS389I is part of the sequence called DYS389II. According to the repeat numbers of alleles per locus, the alleles were categorized into short (25%), medium (50%) and long class (25%) as described by Ge *et al*.^14^, used to evaluate the relationship between allele size and corresponding mutation rate.

## Results and Discussion

### Allele frequencies and gene diversity

Allele frequencies and gene diversity values for each locus are listed in Supplemental Table 1. A total of 190 alleles were detected at 22 Y-STR loci with the allele frequencies ranged from 0.0018 to 0.7676. The number of alleles at each locus ranged from 4 for Y_GATA_H4 to 15 for DYS447. At two multi-copy loci DYS385a/b and DYS527a/b, 71 allelic combinations with 16 separated alleles and 40 allelic combinations with 11 alleles were observed, repectively. Single-copy locus DYS449 and multi-copy locus DYS385a/b showed the highest GD values as 0.8883 and 0.9658, respectively. Except DYS391 and DYS438, the GD values for the other 20 loci were all obove 0.5, which suggests high polymorphisms in the Northern Chinese Han population.

### Haplotype diversity

The haplotype distribution in a sample of 539 unrelated Northern Chinese Han males for the 22 Y-STRs is shown in the Supplementary Table 2. Haplotype diversity and forensic parameter based on various combinations of Y-STRs, such as minimal haplotype, extended haplotype, Y filer and this study are shown in Table 1. Within these unrelated 539 Northern Chinese Han individuals, a total of 537 haplotypes were observed at the 22-loci resolution and the haplotype diversity value of was 0.99998. 535 haplotypes (99.63%) were observed once and only 2 were observed twice (0.37%). 492 haplotypes were observed at the minimal haplotype STRs resolution (DYS19, DYS389I/II, DYS390, DYS391, DYS392, DYS393, and DYS385). For the extended haplotype STRs (minimal haplotype STRs, DYS438 and DYS439), 517 haplotypes were observed.

**Table 1:** Haplotype diversity and forensic parameters for different combinations of Y-STRs in 539 unrelated Northern Chinese Han.

No. of observed haplotypes	Combined Y-STRs			
	Minimal haplotype	Extended haplotype	Y filer	This study
1(unique)	458	500	531	535
2	26	13	4	2
3	4	3	0	0
4	3	1	0	0
5	1	0	0	0
Number of haplotypes	492	517	535	537
Unique haplotypes	458	500	531	535
Fraction of unique haplotypes	93.09%	96.71%	99.25%	99.63%
Discrimination capacity	0.999537	0.999798	0.999963	0.999977
Haplotype diversity ± SD	0.9995 ± 0.0002	0.9998 ± 0.0001	1.0000 ± 0.0001	1.0000 ± 0.0001

In the case of 17 Y-STRs (extended haplotype STRs, DYS437, DYS448, DYS456, DYS458, DYS635, and GATA H4.1) from AmpFlSTR® Yfiler™ kit, 535 haplotypes were observed with the haplotype diversity value of 0.99996. From these, 531 haplotypes (99.25%) were observed once, 4 were observed twice (0.75%). Although the number of unique haplotype increased when additional Y-STR loci were combined, however, in this study, only 2 unique haplotype were increased with 5 loci were added compared with Y filer. This suggest that to achieve the goal for high haplotype resolution for Y-STR analysis, selecting appropriate loci, such as the Rapidly mutating Y-STRs^15^, should be considered.

### Variant alleles

Thirty four copy number variants were detected in 1123 males. Variant alleles were confirmed by re-amplification and genotyping. Null alleles were observed at DYS448 (6 father–son pairs), DYS19 (1 father–son pair) and DYS527a/b (1 father–son pair). Primers were designed for larger PCR fragments of these 3 loci, but failed to produce amplicons in the test samples (data not shown). DYS448 is located within the azoospermia factor c gene (AZFc) in the distal euchromatic part of the Y chromosome long arm. AZFc consists almost entirely of very long direct and inverted repeats. Therefore, it is prone to partial deletions or duplications by rearrangements^16^. The DYS448 null allele has been reported by several studies^17–20^. The relatively high frequencies of the DYS448 null allele in Asians suggest giving careful consideration to the use of DYS448 for commercial genotyping and further database construction in Asians. Triplications were observed at DYS527a/b (8 father–son pairs) and DYS385 a/b (1 father–son pair). These variants are not rare in forensic casework and they should be interpreted carefully to exclude mixed profiles. These variants have been considered due to non-allelic, homologous recombination^21^.

### Mutation rates

In this study, 578 meiosis from fathers to sons were observed, in which 47 mutations were found at all the studied loci except DYS47, DYS438, DYS447, DYS522, and DYS388 (Table 2). There are no more than one locus mutations in the same father-son pair. Except one three-step mutation occurred at DYS449 (32 → 29), all remaining mutations were single step, namely, 97.9% mutations were one step. This finding is consistent with the general notion that the majority of mutations comprise single step repeat gain or loss due to strand slippage during replication^22^. Among these 47 mutations, 26 mutations (i.e., 55.3%) gained repeats, and 21 mutations (i.e., 44.7%) lost repeats. Hence, the data herein support that mutations at these Y chromosome microsatellites do not have any contraction or expansion bias.

**Table 2:** The mutation and rates for the 22 Y-STR loci studied in Northern Chinese Han.

Locus	No. of meiosis	Mutation count	No. of mutations with repeat gains	No. of mutations with repeat losses	Mutation rate × 10^**−3**^	95% CI × 10^**−3**^
DYS19	578	1		1	1.7	0–9.6
DYS385a/b	1156	4	2	2	3.5	0.9–8.8
DYS389I	578	2	1	1	3.5	0.4–13.2
DYS389II	578	5	3	2	8.7	0.3–21.3
DYS390	578	3	2	1	5.2	1.1–16.0
DYS391	578	1	1		1.7	0–10.2
DYS392	578	1	1		1.7	0–10.2
DYS393	578	1	1		1.7	0–10.2
DYS437	578	0			0	0–6.4
DYS438	578	0			0	0–6.4
DYS439	578	4	4		6.9	1.9–17.6
DYS448	578	1		1	1.7	0–9.6
DYS456	578	1	1		1.7	0–10.2
DYS458	578	5	3	2	8.7	0.3–21.3
DYS635	578	1	1		1.7	0–10.2
Y_GATA_H4	578	1	1		1.7	0–10.2
DYS449	578	9	4	5	15.6	7.6–31.1
DYS447	578	0			0	0–6.4
DYS522	578	0			0	0–6.4
DYS388	578	0			0	0–6.4
DYS444	578	2		2	3.5	0.4–13.2
DYS527a/b	1156	5	1	4	4.3	1.4–10.1
Summary	13872	47	26	21	3.4	2.5–4.5

The average mutation rate across these 22 Y-STR loci was 0.0034 (95% confidence interval (CI), 0.0025–0.0045), which was close to the average mutation rates across 16 Y-STR markers of the Texas populations (i.e., 0.0021) by Ge *et al*.^14^ and the South China Han population (i.e., 0.0023) by Weng *et al*.^23^ The mutation rates of the 22 Y-STR loci ranged from 0.0000 (95% CI, 0.0000–0.0064) to 0.0156 (95% CI, 0.0076–0.0311). Mutation counts and rates by relative allele sizes (short, moderate, and long) for each locus is shown in Table 3. In the Northern Chinese Han population, the mutation rate of long alleles (6.9 × 10^−3^) is significantly greater than short (1.9 × 10^−3^) and moderate (2.5 × 10^−3^) alleles. Therefore, the longer alleles are more likely to be mutated than short alleles, which is consistent with the previous studies^14,23,24^.

**Table 3:** Mutation counts and rates by relative allele sizes (short, moderate, and long) for each locus.

Locus	Motif	Allele range	Short	Moderate	Long
DYS19	TAGA	10–19	1		
DYS385a/b	GAAA	7–28		4	
DYS389I	(TCTG)(TCTA)	9–17		2	
DYS389II	(TCTG)(TCTA)(TCTG)(TCTA)	24–34			5
DYS390	(TCTA)(TCTG)	17–28		3	
DYS391	TCTA	6–14	1		
DYS392	TAT	6–17	1		
DYS393	AGAT	9–17		1	
DYS437	TCTA	13–17			
DYS438	TTTTC	6–14			
DYS439	AGAT	9–14	1	3	
DYS448	AGAGAT	20–26			1
DYS456	AGAT	13–18		1	
DYS458	GAAA	13–20		2	3
DYS635	T**S**TA compound	17–27		1	
Y_GATA_H4	TAGA	8–13	1		
DYS449	TTTC	26–36			9
DYS447	TAA**W**A compound	22–29			
DYS522	GATA	8–17			
DYS388	ATT	10–18			
DYS444	TAGA	11–15	1	1	
DYS527a/b	(GAAA)(GGAA)	15–28			5
No. of mutations			6	18	23
No. of alleles			3200	7346	3326
Mutation rate × 10^−3^			1.9	2.5	6.9
95% CI × 10^−3^			0.7–4.1	1.5–3.9	4.4–10.4

The allele sizes are generally categorized into 25%, 50%, and 25% quantiles for short, moderate and long allele sizes, respectively.

It is more accurate to estimate the Y-STR mutation rate is by testing a large number of meiosis from father-son pairs. Ballantyne *et al*.^25^ provided Y-STR mutation rates for a large number of Y-STR markers in a reasonably large number of up to 2000 DNA-confirmed father-son pairs collected from the Germany and Poland. Burgella *et al*.^26^ performed a meta-analysis to estimate the mutation rate for 110 Y-STRs combining population and father–son pair data. A comparison of our data to these published rates was shown in Table 4. The mutation rates for most of the shared loci were similar except DYS449, which was 1.9 × 10^−3^ reported by Burgarella and only approximately one eighth and one seventh of our and Ballantyne’s study.

**Table 4:** Comparison between the mutation rates of this study and other two published studies with large number of meiosis.

Locus	This study				Ballantyne *et al*.^25^				Burgarella *et al*.^26^			
	No. of meiosis	Mutation count	Mutation rate × 10^−3^	95% CI × 10^**−3**^	No. of meiosis	Mutation count	Mutation rate × 10^**−3**^	95% CI × 10^**−3**^	No. of meiosis	Mutation count	Mutation rate × 10^**−3**^	95% CI × 10^**−3**^
DYS19	578	1	1.7	0–9.6	1756	7	4.4	2.0–8.2	14632	32	2.2	1.6–3.1
DYS385 a/b*	1156	4	3.5	0.9–8.8	1762	3	2.1	0.6–5.1	/	/	/	/
					1615	6	4.1	1.8–8.1				
DYS389 I	578	2	3.5	0.4–13.2	1751	9	5.5	2.7–9.7	12651	32	2.5	1.8–3.6
DYS389 II	578	5	8.7	0.3–21.3	1743	6	3.8	1.6–7.5	/	/	/	/
DYS390	578	3	5.2	1.1–16.0	1758	2	1.5	0.4–4.1	14131	30	2.1	1.5–3.0
DYS391	578	1	1.7	0–10.2	1759	5	3.2	1.3–6.7	13995	38	2.7	1.8–3.7
DYS392	578	1	1.7	0–10.2	1728	1	9.7	0.1–3.2	13948	6	0.4	0.2–0.9
DYS393	578	1	1.7	0–10.2	1750	3	2.1	0.6–5	12576	13	1	0.6–1.8
DYS437	578	0	0	0–6.4	1760	2	1.5	0.4–4.1	9238	10	1.1	0.6–2
DYS438	578	0	0	0–6.4	1751	1	1	0.1–3.2	9339	4	0.4	0.2–1.1
DYS439	578	4	6.9	1.9–17.6	1736	6	3.8	1.6–7.5	9313	51	5.8	4.2–7.2
DYS448	578	1	1.7	0–9.6	1747	0	0	0.01–2.1	6655	11	1.7	0.9–3
DYS456	578	1	1.7	0–10.2	1757	8	4.9	2.4–9	6664	30	4.5	3.2–6.4
DYS458	578	5	8.7	0.3–21.3	1756	14	8.4	4.8–13.4	6684	46	6.9	5.2–9.2
DYS635	578	1	1.7	0–10.2	1732	6	3.9	1.6–7.6	7434	23	3.1	2.1–4.6
Y_GATA_H4	578	1	1.7	0–10.2	1755	5	3.2	1.3–6.6	7618	21	2.8	1.8–4.2
DYS449	578	9	15.6	7.6–31.1	1617	19	12.2	7.5–18.5	369	7	1.9	9.2–38.6
DYS447	578	0	0	0–6.4	1722	3	2.1	0.6–5.1	658	3	4.6	1.6–13.3
DYS522	578	0	0	0–6.4	1620	1	1	0.2–3.4	555	0	0	0–6.9
DYS388	578	0	0	0–6.4	1635	0	0	0.02–2.3	2394	1	0.4	0.02–2.4
DYS444	578	2	3.5	0.4–13.2	1775	9	5.5	2.7–9.7	80	0	0	0–45.8
DYS527 a/b	1156	5	4.3	1.4–10.1	/	/	/	/	/	/	/	/

*The mutation rate for DYS385 a/b was calculated separatly as DYS385 a and DYS385 b by Ballantyne’s study.

^/^The data were absent due to these loci were not included in the study.

## Conclusion

In this study, we investigated the haplotype diversity and estimated mutation rates for 22 Y-STRs in 578 father–son pairs in a Northern Chinese Han population. We detected 537 distinct haplotypes in 539 male individuals, which indicating a high power to distinguish unrelated male individuals. Furthermore, totally 47 mutations were observed during 13,872 paternal meiosis. The mutation rate for each locus estimates ranged from 0.0 to 15.6 × 10^−3^ with an average mutation rate 3.4 × 10^−3^ (95% CI 2.5–4.5 × 10^−3^). This study adds to the growing data on Y-STR haplotype diversity and mutation rates. It could be very useful for population and forensic genetics. However, to obtain precise knowledge of haplotype and mutation rate, more number of meiosis analyses involving more Y-STRs loci should be performed.

## Electronic supplementary material

Supplementary table 1

Supplementary table 2
